# Flow cytometric characterization of cecal appendix lymphocyte subpopulations in children: a pilot study

**DOI:** 10.1007/s00383-023-05558-z

**Published:** 2023-09-22

**Authors:** Javier Arredondo Montero, Andrea Torres López, Guillermina Hurtado Ilzarbe, Giuseppa Antona, Raquel Ros Briones, Natalia López-Andrés, Nerea Martín-Calvo

**Affiliations:** 1 Pediatric Surgery Department, Hospital Universitario de Navarra, Calle Irunlarrea 3, 31008 Pamplona, Navarra Spain; 2 Preventive Medicine and Public Health Department, School of Medicine, University of Navarra, Pamplona, Navarra Spain; 3 Hematology and Haemotherapy Department, Hospital Universitario de Navarra, Pamplona, Navarra Spain; 4 Cardiovascular Translational Research, NavarraBiomed (Miguel Servet Foundation), Hospital Universitario de Navarra, Universidad Pública de Navarra (UPNA), IdiSNA, Pamplona, Navarra Spain; 5 Instituto de Investigación Sanitaria de Navarra (IdiSNA), Pamplona, Navarra Spain; 6 CIBER Fisiopatología de la Obesidad y Nutrición (CIBERobn), Health Institute Carlos III, Madrid, Spain

**Keywords:** Flow cytometry, Pediatric acute appendicitis, B-lymphocytes, NK-lymphocytes, T-lymphocytes

## Abstract

**Introduction:**

Scientific literature regarding the characterization of lymphocyte subpopulations of the cecal appendix is sparse, with few precedents limited to immunohistochemical techniques.

**Methods:**

We conducted a prospective pilot study to characterize lymphocyte subpopulations of the cecal appendix in children. Participants were divided into three groups: (1) patients without histological acute appendiceal inflammation, (2) patients with histological uncomplicated acute appendicitis, and (3) patients with histological complicated acute appendicitis (gangrenous, perforated). A fresh sample of the base of the appendix was taken from all patients and a flow cytometric study was performed. Quantitative variables were compared using Kruskal–Wallis test and Mann–Whitney *U* test.

**Results:**

This study included 57 patients divided into Group 1 (*n* = 5), Group 2 (*n* = 37), and Group 3 (*n* = 15). Median values (IQR) of the percentage of B-lymphocytes were 67.8 [66.8–68.1] in group 1, 61.15 [53.74–66.4] in group 2, and 52.1 [33–62.02] in group 3 (*p* = 0.02). Median values (IQR) of the percentage of NK-lymphocytes were 0.26 [0.2–0.3] in group 1, 0.55 [0.37–0.66] in group 2, and 0.84 [0.35–1.45] in group 3 (*p* = 0.008). Median values (IQR) of the percentage of T-lymphocytes were 31.9 [31.7–33.1] in group 1, 37.68 [32.15–45.69] in group 2, and 46.9 [37.03–67] in group 3 (*p* = 0.02). Pair comparisons of groups 2 and 3 also showed significant differences in the percentage of B lymphocytes (*p* = 0.03) and NK-lymphocytes (*p* = 0.02).

**Conclusions:**

Significant differences in lymphocyte subpopulations were identified according to the histologic grade of the cecal appendix. More specifically, a lower percentage of B-lymphocytes and a higher percentage of T- and NK-lymphocytes were observed in cases of acute appendicitis. These findings must be confirmed and their etiopathogenic, diagnostic, and prognostic implications elucidated in future studies with larger sample sizes.

**Supplementary Information:**

The online version contains supplementary material available at 10.1007/s00383-023-05558-z.

## Introduction

Acute appendicitis is a highly prevalent pathology and one of the main reasons for urgent abdominal surgical consultations worldwide. Despite the advances in its management in recent decades, especially with the advent of minimally invasive techniques, many elements regarding acute appendicitis, including its etiopathogenesis, have not been elucidated yet [1, 2]. The development of acute appendicitis has been classically attributed to an obstruction of the appendicular lumen (at the expense of a coprolite or appendicolith), which induces a parietal pressure increase and decreases tissue perfusion capacity, potentially leading to gangrene and perforation. However, this mechanism is not observed in all cases [1]. It has also been proposed that appendiceal inflammation may be secondary to a tumor, a foreign body or an infection (viral or bacterial) [3–5].

Recent studies hypothesized that acute appendicitis may have an allergic trigger. Large retrospective cohort studies showed a protective effect of a history of allergy on the development of complicated appendicitis [6]. On the other hand, recent works showed an increase of IgE cells [7–9], as well as a predominance of eosinophilic infiltration [10–12] in phlegmonous (uncomplicated) appendicitis but not in complicated appendicitis, which reinforces the idea that uncomplicated appendicitis may be immune-mediated by an allergic-type I hypersensitivity reaction. Other studies suggested the existence of distinct immunologic patterns for complicated and uncomplicated acute appendicitis, raising the possibility that they are nosologically distinct entities [13, 14]. This is especially relevant given the current tendency to consider surgical treatment as the first line of therapy in complicated acute appendicitis and to consider non-surgical management in non-complicated appendicitis [1, 2]. Understanding acute appendicitis and its different subtypes from an etiopathogenic and pathophysiologic point of view is essential to adequately direct these therapeutic lines.

To our knowledge, the only study that evaluated lymphocyte subpopulations in the inflamed cecal appendix was published in 2000, was conducted in a mixed population and mainly used immunohistochemical techniques [15]. We hypothesize that the characterization of lymphocyte subpopulations by an accurate method could contribute to a better understanding of this pathology in immunological terms. Therefore, this study aimed to characterize lymphocyte subpopulations using flow cytometry in a fresh sample of the cecal appendix in children.

## Materials and methods

This study was approved by our center's Clinical Research Ethics Committee under code PI_2020/112. The ethical principles of the Declaration of Helsinki were applied to the conduct of this research study. All participants’ parents or legal representatives signed an informed consent form.

### Study design

This study, which constitutes an extension of the BIDIAP cohort [16, 17], is a prospective, observational study aimed to characterize lymphocyte subpopulations of the cecal appendix in children using flow cytometry. Three groups of pediatric patients were included in this study: (1) patients in whom appendectomy was performed and in whom the histological report revealed an absence of acute appendiceal inflammation. This group included patients with suspected acute appendicitis, patients in whom an incidental appendectomy was performed in the context of other acute abdominal pathology, and patients in whom a delayed or interval appendectomy was performed. (2) patients with histologically uncomplicated acute appendicitis (congestive, phlegmonous, or suppurative appendicitis). In addition, (3) patients with histologically complicated acute appendicitis (gangrenous or perforated appendicitis). Congestive appendicitis was considered polymorphonuclear infiltration of the appendix without invasion of the lamina propria. Phlegmonous or suppurative appendicitis was considered polymorphonuclear infiltration of the appendix with invasion of the lamina propria. Gangrenous appendicitis was considered polymorphonuclear infiltration of the appendix with invasion of the lamina propria and/or mural necrosis. Perforated appendicitis was considered to be any of the previous alterations with the presence of micro or macroscopic perforation of the appendix. The histologic study of the samples was performed in all cases by the same senior pathologist, who was blinded to the results of the study. Sociodemographic, clinical, analytical, surgical, radiological, and histological variables of all patients were extracted from participants’ clinical records. Patients were recruited when the personnel conducting the investigation were available at the center. Inclusion and exclusion criteria are shown in Supplementary File 1.

### Sample collection and measurement of lymphocyte subpopulations

During appendectomy, a full-thickness appendiceal transverse section of approximately 0.5 cm in length was taken at the appendico-cecal confluence in all patients. Sections were stored in serum and refrigerated until processing, which was done within 24 h of collection by personnel from the Hematology Department blinded to the patient’s group.

For the flow cytometry study, cell labeling was performed with BD Multitest™ 6-color TBNK reagent (CD3 FITC/CD16 PE + CD56 PE/CD45 PerCP-Cy™5.5/CD4 PE-Cy™7/CD19 APC/CD8 APC-Cy™7). Samples were processed on BD FACSCanto™ II flow cytometers and the analysis were performed with the Infinicyt™Software BD Biosciences.

### Statistical analysis

For descriptive purposes, means and standard deviations or median and interquartile ranges were used for quantitative variables and proportions for categorical ones. Kolmogórov–Smirnov test was used to assess the normality of quantitative variables. Sociodemographic and clinical variables were compared between groups using Kruskal–Wallis test, Fisher’s exact testand Mann–Whitney *U* test. Statistical significance was settled in a *p* value < 0.05 (two tails). Statistical analysis was performed with STATA 17.0 (StataCorp LCC).

## Results

### Demographic and clinical characteristics

This study included 57 patients divided into 3 groups: (1) patients who underwent an appendectomy and in whom the histological report revealed an absence of acute appendiceal inflammation (*n* = 5). This group included patients with suspected acute appendicitis (*n* = 1), patients who underwent an incidental appendectomy in the context of other acute abdominal pathology (*n* = 2), and patients who underwent a delayed or interval appendectomy (*n* = 2). (2) Patients with histological uncomplicated acute appendicitis (NCAA) (i.e., congestive, phlegmonous, or suppurative appendicitis) (*n* = 37). (3) Patients with histological complicated acute appendicitis (CAA) (i.e., gangrenous or perforated appendicitis) (*n* = 15). Nine participants were excluded from the analyses due to sample processing errors (2 patients from Group 1, 5 patients from Group 2, and 2 patients from Group 3). These errors included: (1) delay in processing of more than 24 h, (2) failure to refrigerate the sample before processing, (3) loss of the sample. No significant differences were found in sociodemographic and clinical characteristics between included and excluded patients (data not shown). Patients in group 3 presented a significantly higher number of emetic episodes (*p* = 0.03) and a higher level of serum C-reactive protein (*p* = 0.002). Participants’ sociodemographic and clinical characteristics by group are shown in Table 1.

**Table 1:** Sociodemographic and clinical characteristics of the study patients

Sociodemographic and clinical variables	Group 1 (absence of acute appendiceal inflammation) *N* = 5	Group 2 (NCAA) *N* = 37	Group 3 (CAA) *N* = 15	*p* value
Age (years)	9.8 (3.2)	10 (2.7)	10.2 (2.8)	0.99*
Sex (male/female) (male %)	3/2 (60%)	24/13 (64.9%)	9/6 (60%)	0.91*
Hours of pain evolution	–	32.7 (24.7)	48.7 (32)	0.06
Number of emetic episodes	–	1.4 (2)	2.6 (2)	**0.03**
Presence of fever at home prior to ED admission (yes/no) (yes %)	–	12/25 (32.4%)	7/8 (46.7%)	0.36
Peripheral venous blood absolute lymphocyte count (1 × 10^9^/L)^a^	–	1.7 [1.2–2.3]	1.2 [0.6–1.9]	0.09
Peripheral venous blood absolute neutrophil count (1 × 10^9^/L)^a^	–	9.2 [5.9–13.3]	11.5 [8.9–17.3]	0.06
Serum C-reactive protein (mg/L)^a^	–	17.8 [5.1–31.3]	44.8 [27.1–100.1]	**0.002**

Bold is for statistically significant analyses

Numbers are mean (standard deviation) or number (percentage)

*NCAA* non-complicated pediatric acute appendicitis, *CAA* complicated pediatric acute appendicitis, *ED* Emergency Department

^a^Median, IQR

* 3-groups comparison

### Flow cytometric study of the cecal appendix (comparison between the 3 groups)

Median values (IQR) of the percentage of B-lymphocytes were 67.8 [66.8–68.1] in group 1, 61.15 [53.74–66.4] in group 2, and 52.1 [33–62.02] in group 3 (*p* = 0.02). Median values (IQR) of the percentage of NK-lymphocytes values were 0.26 [0.2–0.3] in group 1, 0.55 [0.37–0.66] in group 2, and 0.84 [0.35–1.45] in group 3 (*p* = 0.008). Median values (IQR) of the percentage of T-lymphocytes were 31.9 [31.7–33.1] in group 1, 37.68 [32.15–45.69] in group 2, and 46.9 [37.03–67] in group 3 (*p* = 0.02).

Significant differences were observed in the percentage of B-lymphocytes, NK-lymphocytes, and T-lymphocytes between the three groups (Table 2). B-lymphocyte percentage decreased from group 1 to group 3 (group 1 higher values, group 2 intermediate values, group 3 smaller values) (*p* = 0.02). A similar pattern was seen in the percentage of CD4+ T-lymphocytes, although this analysis did not reach statistical significance (*p* = 0.14). Conversely, the percentage of NK- and T-lymphocytes increased from group 1 to group 3 (group 1 smaller values, group 2 intermediate values, group 3 higher values) (NK, *p* = 0.008; T, *p* = 0.02).

**Table 2:** Flow cytometric study of the patient’s included in the study

Flow cytometry analysis	Group 1 (Absence of acute appendiceal inflammation) *N* = 5	Group 2 (NCAA) *N* = 37	Group 3 (CAA) *N* = 15	*p* value (3-groups comparison)^a^	*p* value (NCAA vs CAA)^b^
B-lymphocytes (%)	67.8 [66.8–68.1]	61.2 [53.7–66.4]	52.1 [33–62]	**0.02**	**0.03**
NK-lymphocytes (%)	0.26 [0.2–0.3]	0.55 [0.37–0.66]	0.84 [0.35–1.45]	**0.008**	0.26
T-lymphocytes (%)	31.9 [31.7–33.1]	37.7 [32.2–45.7]	46.9 [37–67]	**0.02**	**0.02**
TCD4+ lymphocytes (%)	23.1 [20.5–23.4]	27.3 [21–32.1]	36 [19.8–43]	0.14	0.34
TCD8+ lymphocytes (%)	8.4 [6.5–8.7]	7.8 [6.5–11.8]	9.6 [7–15.9]	0.27	0.16
TCD4+/TCD8+ ratio	3.1 [2.2–3.6]	3.1 [2.6–4.1]	2.9 [1.9–3.8]	0.62	0.41
TCD4+CD8+ lymphocytes (%)	0.6 [0.4–0.6]	0.6 [0.3–0.8]	0.6 [0.3–0.9]	0.84	0.63
TCD4−CD8− lymphocytes (%)	1.4 [1.2–1.7]	1.6 [1–2.2]	1.5 [1–3.5]	0.81	0.57

Bold is for statistically significant analyses

Numbers are median, IQR

*NCAA* non-complicated pediatric acute appendicitis, *CAA* complicated pediatric acute appendicitis

^a^Kruskal–Wallis test

^b^Mann–Whitney *U* test

No differences were found in the percentage of TCD8+ lymphocytes (*p* = 0.27), TCD4+CD8+ lymphocytes (*p* = 0.84), TCD4−CD8− lymphocytes (*p* = 0.81) or in the TCD4+/TCD8+ ratio (*p* = 0.62).

**Fig. 1:**
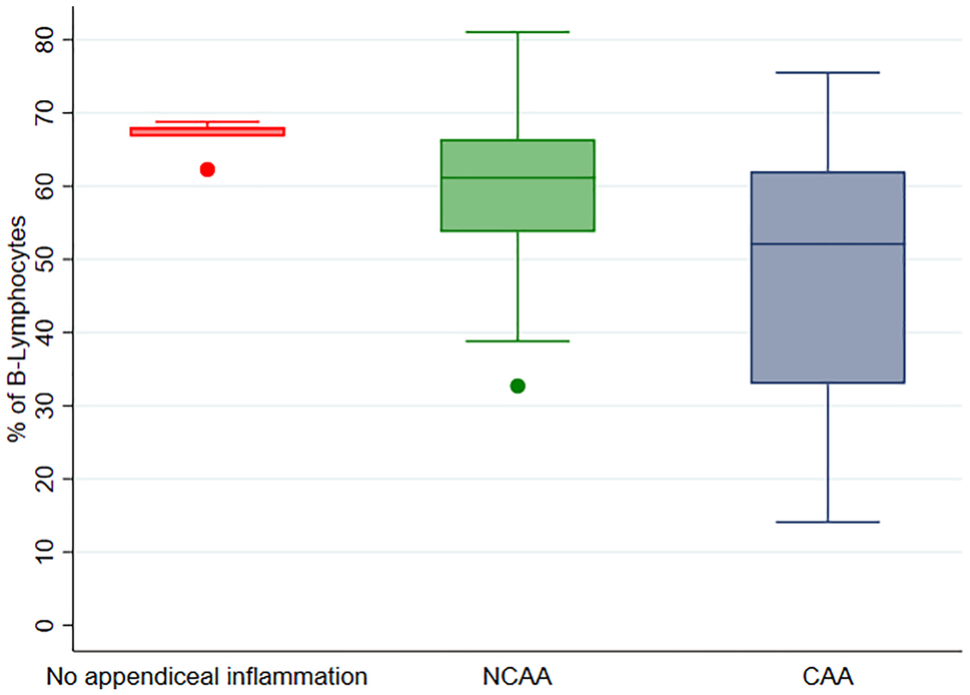
Graphical representation of the percentage of B-lymphocytes by group. *NCAA* non-complicated pediatric acute appendicitis, *CAA* complicated pediatric acute appendicitis

**Fig. 2:**
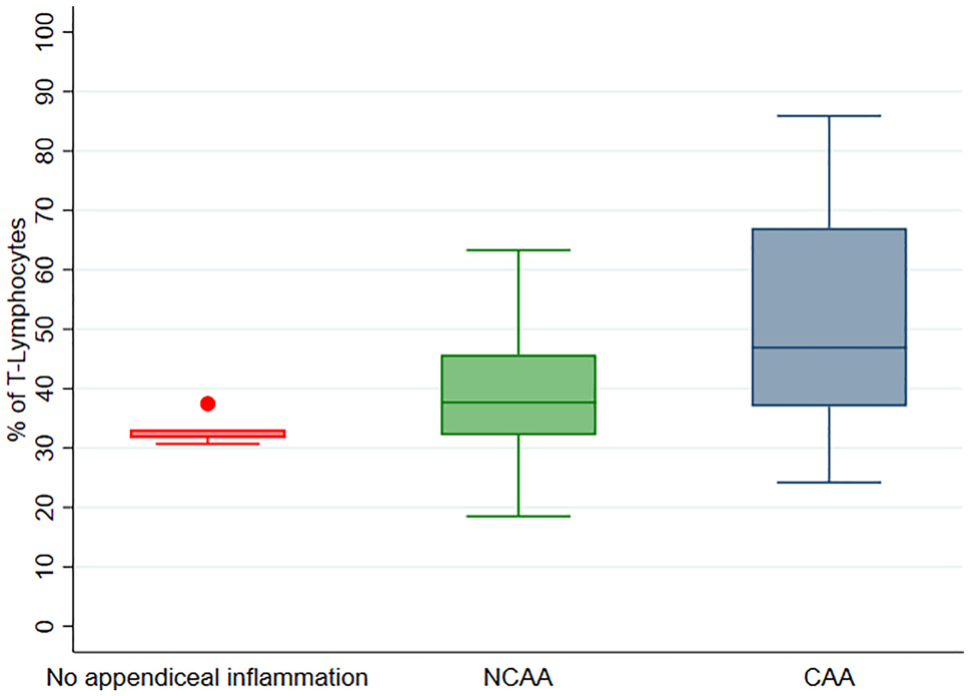
Graphical representation of the percentage of T-lymphocytes by group. *NCAA* non-complicated pediatric acute appendicitis, *CAA* complicated pediatric acute appendicitis

**Fig. 3:**
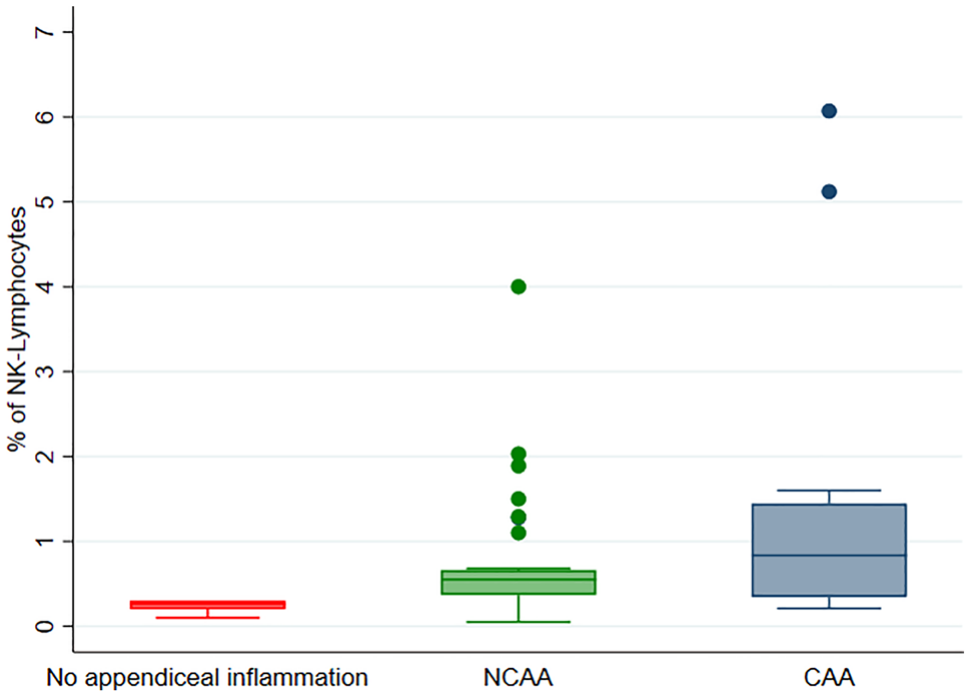
Graphical representation of the percentage of NK-lymphocytes by the group. *NCAA* non-complicated pediatric acute appendicitis, *CAA* complicated pediatric acute appendicitis. Note the presence of outliers in groups 2 (NCAA) and 3 (CAA)

The graphical representation of the percentage of B-, T-, and NK-lymphocytes by group is shown in Figs. 1, 2, and 3.

### Flow cytometric study of the cecal appendix (complicated vs. non-complicated pediatric acute appendicitis)

When we repeated the analyses comparing only the NCAA and CAA groups, we observed a significantly lower mean percentage of B-lymphocytes (*p* = 0.03) and a higher mean percentage of T-lymphocytes in the CAA group (*p* = 0.02) (Table 2). No other significant differences were observed.

Atypical or infrequent histological findings were found in three patients, all belonging to group 2 (NCAA). Cytometric characterization of these patients is described in Supplementary Table 2.

**Fig. 4:**
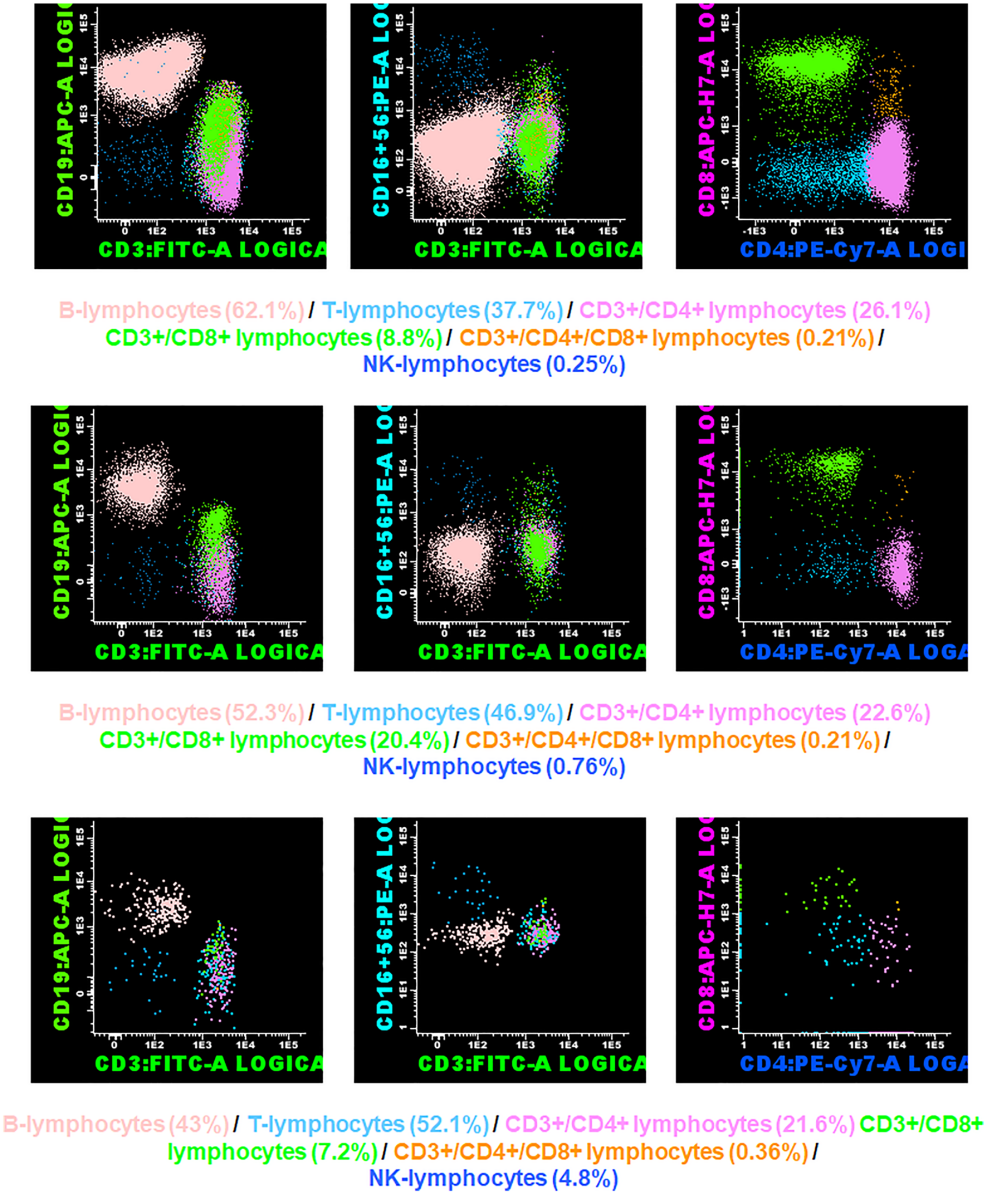
Graphical representation of the flow cytometric analyses performed. Top row: patient from group 1 (absence of inflammation in the cecal appendix). Middle row: patient from group 2 (NCAA). Bottom row: patient from group 3 (CAA)

The graphical depiction of the most representative flow cytometric studies by group is shown in Fig. 4.

## Discussion

In this prospective observational study flow cytometry was used to characterize the distribution of lymphocytes subpopulations in a fresh sample of cecal appendix from 57 children and compare them according to the histological gradation of the tissue. Our results showed differences in the percentage of B-, T-, and NK-lymphocytes when comparing the three groups and in the percentage of B- and T-lymphocytes when comparing the NCAA and CAA subgroups. Specifically, we found a higher percentage of NK- and T-lymphocytes and a lower percentage of B-lymphocytes in the CAA group.

To our knowledge, this is the first study that accurately characterizes appendiceal lymphocyte subpopulations in the absence of inflammation and in the setting of complicated and uncomplicated pediatric acute appendicitis (PAA). Therefore, our results are important, because they can help to understand the immune dynamics in the three scenarios evaluated, which may have potential implications in terms of etiopathogenic understanding, diagnosis and stratification, and prognosis.

A relevant aspect of this study is the sampling at the level of the appendico-cecal confluence (appendiceal base) and not in the most pathologic area of the cecal appendix. We believe that this has two implications: first, it promotes standardization and homogenization of the results and facilitates the reproducibility of the study. Second, this demonstrates that variations in the lymphocyte subpopulations of the different histologic subtypes of PAA are diffuse and not focal, which is a finding of interest for future studies. We hypothesize that there will be a gradient in the distribution of lymphocyte subpopulations from the most pathologic area of the appendix (focal perforation, focal gangrene) to the appendico-cecal confluence, but this theory has not been proven so far. Future comparative studies evaluating lymphocyte subpopulations in the perforation and at the appendico-cecal confluence are needed.

Regarding appendiceal tisular topography, current processing for flow cytometry involves smashing the tissue and processing it *en bloc*. However, we believe that since appendicitis is a process that selectively affects different histological layers depending on its histological subtype, it would be interesting to consider the possibility of performing a specific flow cytometry study of the mucosa and serosa separately (the layers that can be more easily separated from the rest).

Our results agree with previous evidence by Kuga et al. 15, who studied 27 patients through immunohistochemical techniques and characterized 12 of them with peripheral blood flow cytometry. They found a higher presence of CD8+ lymphocytes and NK lymphocytes in the histological analysis of CAA patients compared with NCAA patients but a lower number of NK-lymphocytes in their peripheral blood flow cytometry. In our study, no differences were found in the TCD8+ lymphocyte subpopulations, but we did find a significantly higher percentage of NK-lymphocytes in CAA patients compared with NCAA ones. It is noteworthy that Kuga et al. divided the cecal appendix into two equal fragments and did not specifically analyze the appendico-cecal confluence as we did. Regarding the analytic technique, it is known that flow cytometry allows for better quantification of lymphocyte subpopulations than immunohistochemistry. Also, it should be mentioned that the study by Kuga et al. included both adult and pediatric Asian populations, which may imply important sociodemographic and clinical differences compared with the population included in our study.

Another interesting question is whether these variations in lymphocyte subpopulations correspond to a focal or systemic process, and what is the degree of correlation between these changes and the time of evolution of symptoms. Given our findings and the precedent set by Kuga et al., we consider comparative analysis between appendiceal flow cytometry and peripheral blood flow cytometry to be a promising field for future studies. A better understanding of when and why these changes occur during the development of appendicitis would greatly contribute to better characterizing this pathology, its diagnosis, and treatment.

Finally, this study involves a pilot characterization of lymphocyte subpopulations by flow cytometry, but there are specific analyses within this technique that we have not been able to perform due to technical and economic limitations. For example, measuring the Th1 and Th2 profiles would allow correlation with the immunological profiles previously postulated for appendicitis subtypes [14].

The main strengths of this study are its prospective design and its rigorous statistical methodology. Nevertheless, this work is not free of limitations: (1) convenience recruitment was performed. To avoid a potential selection bias, strict inclusion and exclusion criteria were followed. Along with this, nine participants were excluded due to missing data. Nevertheless, the comparison of sociodemographic and clinical characteristics of the included and excluded participants did not show significant differences. (2) The small sample size of group 1 (*n* = 5) limited the power of the analyses and makes it difficult to extrapolate the results. Likewise, this group of patients is highly heterogeneous, which is a major limitation. (3) PAA constitutes a spectrum, and rigid categorization at the histological level may not be representative. In our case, only a senior pathologist evaluated the specimens. (4) For this study, given its pilot character, the standard flow cytometry protocol of our center was implemented for B, T and NK subpopulations, which did not include the determination of eosinophils. Given the existing precedents in the literature about this cell subpopulation, we believe that it would have been of enormous interest. (5) A directed microbiological study of all patients (stool culture, peritoneal fluid culture) was not obtained regularly. Given that infectious etiologies (acute gastroenteritis, intestinal parasitosis, etc.) should be considered in the differential diagnosis of PAA, we believe that in future studies it would be convenient to perform these analyses exhaustively in all patients.

## Conclusions

In conclusion, we found significant differences in the percentage of lymphocyte subpopulations in the cecal appendix of children without appendiceal inflammation, with NCAA, and with CAA. This is a pilot study, so future multicenter studies with larger sample sizes are needed to validate these findings and better characterize PAA at the immunologic level.

### Supplementary Information

Below is the link to the electronic supplementary material.

Supplementary file1 (DOCX 16 KB)

Supplementary file2 (DOCX 17 KB)

## Data Availability

All data pertaining to this study are available upon justified request through the author in correspondence.
