# Correction to: Flow cytometric characterization of cecal appendix lymphocyte subpopulations in children: a pilot study

**DOI:** 10.1007/s00383-023-05567-y

**Published:** 2023-11-07

**Authors:** Javier Arredondo Montero, Andrea Torres López, Guillermina Hurtado Ilzarbe, Giuseppa Antona, Raquel Ros Briones, Natalia López-Andrés, Nerea Martín-Calvo

**Affiliations:** 1 Pediatric Surgery Department, Hospital Universitario de Navarra, Calle Irunlarrea 3, 31008 Pamplona, Navarra Spain; 2 Preventive Medicine and Public Health Department, School of Medicine, University of Navarra, Pamplona, Navarra Spain; 3 Hematology and Haemotherapy Department, Hospital Universitario de Navarra, Pamplona, Navarra Spain; 4 Cardiovascular Translational Research, NavarraBiomed (Miguel Servet Foundation), Hospital Universitario de Navarra, Universidad Pública de Navarra (UPNA), IdiSNA, Pamplona, Navarra Spain; 5 Instituto de Investigación Sanitaria de Navarra (IdiSNA), Pamplona, Navarra Spain; 6 CIBER Fisiopatología de la Obesidad y Nutrición (CIBERobn), Health Institute Carlos III, Madrid, Spain

**Correction to: Pediatric Surgery International (2023) 39:274** 10.1007/s00383-023-05558-z

In the original publication, authors would like to make corrections in the abstract section and in discussion text.

In the abstract, in the results section, it states: “Pair comparisons of groups 2 and 3 also showed significant differences in the percentage of B lymphocytes (p = 0.03) and NK-lymphocytes (p = 0.02).”. This is an error, since the analyses for NK-lymphocytes (group 2 vs. 3) were non-significant (p = 0.26). The correct sentence would be: “Pair comparisons of groups 2 and 3 also showed significant differences in the percentage of B-lymphocytes (p = 0.03) and T-lymphocytes (p = 0.02).”

In the discussion (page 5), it states: “In our study, no differences were found in the TCD8+ lymphocyte subpopulations, but we did find a significantly higher percentage of NK-lymphocytes in CAA patients compared with NCAA ones.”. This sentence is not correct, since although we did find a higher percentage of NK-lymphocytes in patients with CAA, this analysis did not reach statistical significance (p = 0.26). The correct sentence would be: “In our study, no differences were found in the TCD8+ lymphocyte subpopulations. We did find a higher percentage of NK-lymphocytes in CAA patients compared with NCAA ones, although this analysis did not reach statistical significance.”

