# Supplementary material for: Flow cytometric characterization of cecal appendix lymphocyte subpopulations in children: a pilot study
Source: Pediatr Surg Int. 2023 Sep 22;39(1):274. doi: 10.1007/s00383-023-05558-z (PMC10516785; doi:10.1007/s00383-023-05558-z)
Supplement: Supplementary file 1 — Supplementary file1 (DOCX 16 KB) [file 383_2023_5558_MOESM1_ESM.docx]

**Supplementary file 1. Inclusion and exclusion criteria**

**Inclusion criteria**

Patients aged 0 to 14 years who underwent appendectomy in our center in the period concerning the prospective recruitment of this study.

**Exclusion criteria**

- Explicit refusal by the patient or the patient's parents/legal guardians

- Patients with metastatic neoplasia.

- Patients with hematological alterations.

- Patients with active autoimmune disorders.

- Patients previously appendectomized.

- Patients who have been treated with immunosuppressants in the 28 days prior to the inclusion.

- Patients who have been treated with systemic steroids in the 14 days prior to the inclusion.

- Patients who have had abdominal trauma prior to the inclusion.
