# Supplementary material for: Flow cytometric characterization of cecal appendix lymphocyte subpopulations in children: a pilot study
Source: Pediatr Surg Int. 2023 Sep 22;39(1):274. doi: 10.1007/s00383-023-05558-z (PMC10516785; doi:10.1007/s00383-023-05558-z)
Supplement: Supplementary file 2 — Supplementary file2 (DOCX 17 KB) [file 383_2023_5558_MOESM2_ESM.docx]

**Supplementary file 2. Patients with infrequent histological findings included in the study**

| **Patient No.** | **Group** | **Histological findings** | **B Lymphocytes %** | **T Lymphocytes %** | **NK Lymphocytes %** | **TCD4+ Lymphocytes %** | **TCD8+ Lymphocytes %** | **TCD4+CD8+ Lymphocytes %** | **TCD4-CD8- Lymphocytes %** | **CD4+/CD8+**  **ratio** |
| --- | --- | --- | --- | --- | --- | --- | --- | --- | --- | --- |
| 1 | 2 (NCAA) | Parasitic granuloma | 62.02 | 37.03 | 0.87 | 24.76 | 9.49 | 1.26 | 1.52 | 2.61 |
| 2 | 2 (NCAA) | Enterobius Vermicularis infestation | 59.2 | 40.4 | 0.4 | 32.1 | 6.5 | 1.3 | 0.7 | 4.94 |
| 3 | 2 (NCAA) | Granulomas  (IBD suspicion) | 65.19 | 35.51 | 0.3 | 27.02 | 6.07 | 0.37 | 1.05 | 4.45 |

IBD: inflammatory bowel disease
